# Using a Fragment-Based Approach To Target Protein–Protein Interactions

**DOI:** 10.1002/cbic.201200521

**Published:** 2013-01-23

**Authors:** Duncan E Scott, Matthias T Ehebauer, Tara Pukala, May Marsh, Tom L Blundell, Ashok R Venkitaraman, Chris Abell, Marko Hyvönen

**Affiliations:** [a] Department of Chemistry, University of CambridgeLensfield Road, Cambridge, CB2 1EW (UK); [b] Department of Biochemistry, University of Cambridge80 Tennis Court Road, Old Addenbrooke's Site, Cambridge, CB2 1GA (UK) E-mail: mh256@cam.ac.uk; [c] Hutchison/MRC Research Centre, University of CambridgeHills Road, Cambridge, CB2 0XZ (UK)

**Keywords:** DNA repair, drug design, fragment-based drug discovery, protein–protein interactions, RAD51

## Abstract

The ability to identify inhibitors of protein–protein interactions represents a major challenge in modern drug discovery and in the development of tools for chemical biology. In recent years, fragment-based approaches have emerged as a new methodology in drug discovery; however, few examples of small molecules that are active against chemotherapeutic targets have been published. Herein, we describe the fragment-based approach of targeting the interaction between the tumour suppressor BRCA2 and the recombination enzyme RAD51; it makes use of a screening pipeline of biophysical techniques that we expect to be more generally applicable to similar targets. Disruption of this interaction in vivo is hypothesised to give rise to cellular hypersensitivity to radiation and genotoxic drugs. We have used protein engineering to create a monomeric form of RAD51 by humanising a thermostable archaeal orthologue, RadA, and used this protein for fragment screening. The initial fragment hits were thoroughly validated biophysically by isothermal titration calorimetry (ITC) and NMR techniques and observed by X-ray crystallography to bind in a shallow surface pocket that is occupied in the native complex by the side chain of a phenylalanine from the conserved FxxA interaction motif found in BRCA2. This represents the first report of fragments or any small molecule binding at this protein–protein interaction site.

## Introduction

Interactions between cellular proteins are crucial to many regulatory functions, playing important roles in signal-transduction pathways, apoptosis and DNA repair.[1] Until recently, disrupting protein–protein interactions with small molecules for chemical biology or the development of therapeutics had been largely neglected. The identification of small molecules that interrupt protein–protein interactions presents unique challenges, mostly due to the nature of the interaction surface.[2] Proteins often interact across large, relatively featureless contact areas (∼750–1500 Å^2^)[3] that lack the grooves, clefts or pockets that define the small-molecule binding sites traditionally targeted for drug discovery.

Alanine-scanning mutation studies, albeit on a relatively small number of protein–protein interfaces, have shown that for some interactions a few surface residues can contribute most of the binding energy.[4, 5] Computational methods such as HSPred[6] are being developed to predict protein–protein interaction (PPI) hotspots and their druggability. Dr PIAS[7, 8] is a web-based service for assessing the druggability of PPIs, and ANCHOR[9] identifies key “anchor” residues at interacting protein surfaces. Pocket prediction has been implemented with algorithms such as Q-SiteFinder[10] and was used in a study by Fuller et al. to demonstrate key differences in the nature of PPIs and other protein–ligand interactions.[11] Hand-curated databases of protein:protein complexes and their inhibitors, such as 2P2IDB[12] and TIMBAL,[13] have been constructed to facilitate the specific study of protein–protein interactions. Further to these, experimental approaches to druggability have been developed based upon a correlation between the physical properties of protein pockets and NMR screening hit rates.[14] The presence of these “hot-spots” on protein interaction surfaces, the lack of selectivity of many active-site targeted inhibitors and the discovery of some potent inhibitors of protein–protein interactions has prompted a re-evaluation of the druggability of this potentially rich pool of targets.

There is no general strategy for screening small molecules that disrupt protein–protein interactions. For example, it is often the case that there is no enzymatic function related to the disruption of the interaction, and so activity-based assays cannot be used. This has focused attention onto the use of a broad range of biophysical techniques such as NMR spectroscopy,[15] surface plasmon resonance (SPR)[16] and X-ray crystallography[17] to verify hit compounds.

These are the same techniques as are employed in fragment-based approaches, which have emerged in structure-based drug design, albeit they are typically used for targeting enzyme active sites.[18, 19] It is thought that the fragments identified as hits in a screen will have a weak affinity that reflects a high intrinsic binding enthalpy overcoming the entropic penalty of binding.[20, 21] As such, fragments can be very efficient ligands and thus provide good starting points for chemical elaboration.[22] However, given the relatively featureless nature of protein surfaces and the general weak potency of fragments, it is not evident that fragments would bind in shallow surface pockets. One strategy that was adopted to address this problem was “tethering” disulfides to a covalently modified cysteine residue suitably located on the surface.[23, 24] The general applicability of tethering is limited in part by the necessity for a suitably positioned cysteine residue with precisely the required geometry to allow a thiol fragment to explore its optimum interaction with the protein, and also by the modest range of disulfide fragments that are commercially available.

Noncovalent binding of proteins on protein surfaces has been detected by ^1^H,^15^N or ^1^H,^13^C HSQC NMR experiments, and has provided invaluable structural information to guide fragment elaboration, that is, in the development of Bcl-X_L_ inhibitors.[25] HSQC NMR screening has also been used successfully to screen fragments against the bromodomains of PCAF[26] and CREB binding protein[27] and against KRas.[28] Such NMR experiments require an assignment of the protein spectra from isotopically labelled protein, which can be time-consuming and costly. In addition, the technique is limited to proteins typically smaller than 30 kDa. Ligand-based NMR experiments such as CPMG,[29] WaterLOGSY[30] and STD[31] use less protein and are quicker to run and process. Although used primarily in fragment-based screens against non-PPI targets,[32–37] an STD NMR fragment screen has been reported recently against GTPase KRas; three fragments that bound in a surface pocket located proximal to the end of an α-helix motif of SOS were identified.[38] Chung et al. have reported the use of a fluorescence anisotropy assay to identify fragments that bind to the BET bromodomains BRD2, BRD3 and BRD4 with approximately 10 μm affinity.[39] In an alternative screening strategy, Parks et al. have reported a methodology exemplified by the change in thermal stabilisation of HDM2 to discover antagonists of the HDM2–p53 interaction.[40, 41] Although the most potent hits demonstrated nanomolar affinity for HDM2, these compounds were relatively large and more akin to hits from a typical high-throughput screening campaign than a fragment screen. Fersht et al. have used a thermal-shift screen in conjunction with a WaterLOGSY NMR screen against a surface cavity that is present in the Y220C mutant of p53, even though this is not strictly a protein–protein interaction.[42]

Here we present a methodology of thermal-shift screening and subsequent competitive NMR screens with fragments performed against a protein–protein interaction. Fragment hits from the initial thermal-shift screen were then biophysically validated by isothermal titration calorimetry (ITC), NMR spectroscopy and X-ray crystallography.

In this work we have targeted the protein–protein interaction between the tumour suppressor BRCA2 and the recombination enzyme RAD51. The interaction is mediated through eight repeated peptide motifs, the BRC repeats, found in an evolutionarily conserved region of BRCA2. Several lines of evidence demonstrate that the BRC repeat–RAD51 interaction is essential in cells for the repair of DNA double-strand breaks by homologous DNA recombination, thus engendering cellular hypersensitivity to radiation and genotoxic drugs.[43] In vitro, the 1127-residue region of human BRCA2 that encodes all eight BRC repeats promotes RAD51-dependent DNA strand-exchange reactions.[44] Recent in vitro evidence suggests that this arises from the differential effects of the BRC repeats in stabilising the binding of RAD51 to single-stranded (ss) DNA, whilst inhibiting the binding of RAD51 to double-stranded (ds) DNA. These opposing effects mutually reinforce one another to promote RAD51-dependent strand exchange.[44–48] Thus, small molecules that interfere with the RAD51–BRC repeat interaction should inhibit DNA repair by homologous DNA recombination, thereby providing a valuable tool for chemical biology and for the possible development of therapeutic agents. To date, no well validated inhibitors or fragments for this site have been reported, despite its potential therapeutic importance.

Pellegrini et al. have reported the crystal structure of the fusion protein of human RAD51 joined by a linker peptide to one of the eight BRC repeats, BRC4 (PDB ID: 1N0W).[49] The structure showed that BRC4 utilises an evolutionarily conserved motif with the amino acid sequence “FXXA” to bind RAD51. BRC4 is in contact with RAD51 over 28 amino acids, with well-defined pockets for the side chains of Phe1524 and Ala1527; this reveals a structural basis for the conservation of this “FXXA” motif. In addition, RAD51 uses this same motif to self-associate, forming various oligomeric structures that are essential for its cellular function. It is worth noting that the FXXA binding site of RAD51 is unlike the deep α-helix binding clefts common to some other protein–protein binding sites, instead the FXXA sequence of the BRC repeats binds in a linear conformation across the RAD51 surface. On the other hand, it has been suggested that proteins that order a continuous region of flexible peptide upon binding might be more druggable than proteins that partner with large preformed globular proteins.[50] In either case, the small pocket that binds only the side chain of the phenylalanine in the FXXA motif represents a significant challenge for any drug-discovery programme. The phenylalanine pocket is predicted to be important for ligand binding,[51] and running Q-SiteFinder,[10] ANCHOR[9] and Dr PIAS[7, 8] on the RAD51:BRC4 structure identified the phenylalanine pocket as a high-scoring binding site.

## Results

### Engineering an archaeal surrogate for human RAD51

**Figure 1:**
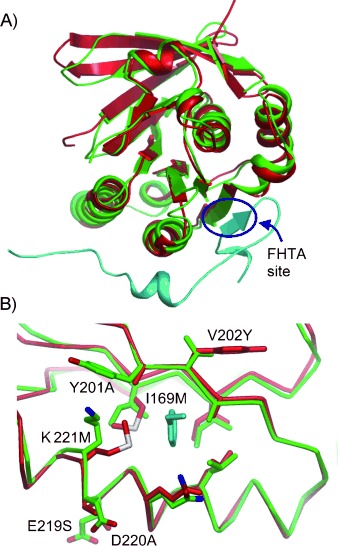
A) Structural overlay of human RAD51 (red) bound to a BRC4 repeat (cyan; PDB ID: 1N0W) and wild-type monomeric *P. furiosus* RadA (green, PDB ID: 1PZN). B) Highlight of the FXXA binding pocket indicating the six mutations that were introduced into humanised RadA. The phenyl group of the FHTA sequence of BRC4 (cyan) is shown in the Phe pocket for reference.

Given the low potency of fragment hits in general, it is essential that any screening is carried out against unliganded protein with an accessible FXXA binding site. The RAD51–BRC4 complex structure was determined by using a covalent fusion construct between the fourth BRC repeat of BRCA2 and the C-terminal ATPase domain of RAD51.[49] Several attempts to generate a monomeric RAD51 by removing the fused BRC4 peptide from this construct or to weaken the self-association by mutagenesis resulted in highly unstable RAD51 protein. Consequently an alternative strategy using the RAD51 orthologue RadA from the thermophilic organism *Pyrococcus furiosus* was employed. Given its high sequence and structural similarity to human RAD51 (Figure 1 A), RadA was anticipated to be a suitable surrogate for the human enzyme for the purpose of fragment-based inhibitor design. Shin et al. have previously crystallised full-length wild-type archaeal RadA in a oligomeric form and shown that, when suitably humanised, it can bind BRC repeats and form nuclear foci in human cells in a BRCA2-dependent fashion similar to human RAD51.[52] Consequently the N-terminal domain of RadA, which contains the self-associating FXXA sequence, was removed to prevent RadA filament formation, and the resulting monomeric protein with an exposed FXXA binding region was found to be stable and suitable for screening.

**Figure 2:**
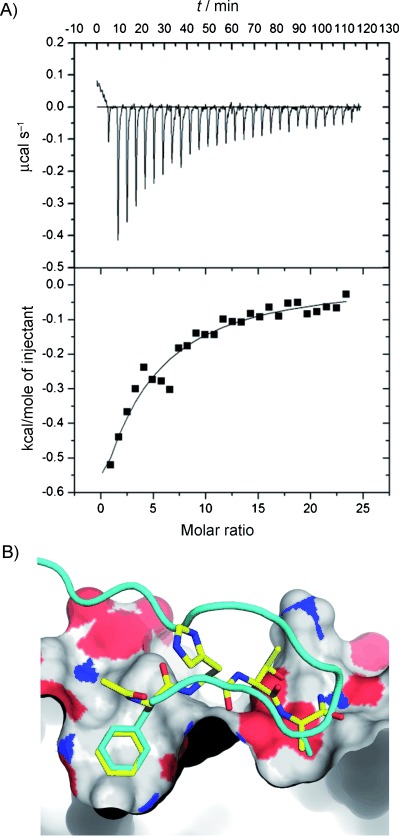
A) FHTA binding to MAYSAM RadA measured by ITC, *K*_d_= (250±50) μm. B) X-ray structure of FHTA (yellow) in complex with MAYM RadA (PDB ID: 4B3B). MAYM RadA was superimposed on human RAD51 (1N0W) and the BRC4 peptide backbone, shown as a cyan ribbon, with the Phe1524 and Ala1527 side chains shown as sticks.

From a comparison of the crystal structures of RAD51 and RadA in the immediate vicinity of the FXXA binding pocket, six key residues were identified that differed between the two proteins (Figure 1 B). Five surface residues Tyr201, Val202, Glu219, Asp220 and Lys221, which are located around the rim of the phenylalanine binding pocket, and Ile169, which forms the base of the pocket, were all mutated to the corresponding residue found in RAD51 in order to humanise the binding pocket. This “humanised” monomeric mutant of RadA is henceforth referred to as “MAYSAM” RadA. Precise details of the humanisation will be reported elsewhere (M.M. et al., unpublished results). The dissociation constant of the FHTA tetrapeptide (FXXA motif of BRC4) for the humanised MAYSAM RadA mutant was measured by ITC to be (250±50) μm, similar to that of wild-type RadA (170 μm). The quadruple mutant “MAYM”, which lacks the E219S and D220A mutations, the side chains of which point away from the Phe pocket and do not contribute to the shape of the Phe pocket, was found to crystallise more readily in a form suitable for compound soaking and was used for all subsequent crystallographic work. As a further validation of the surrogate system, crystals of humanised MAYM RadA were soaked with the tetrapeptide FHTA, and the structure was determined at high resolution. As expected, the FHTA peptide bound in the FXXA binding region of the protein. A superposition of the MAYM RadA:FHTA complex with the crystal structure of human RAD51:BRC4 complex (PDB ID: 1N0W) reveals a good degree of overlap between the two ligands, with similar interactions between the peptide and the protein (Figure 2). These data confirm that the FHTA tetrapeptide can mimic the key interaction between RAD51 and BRCA2, and that it can be used as a site-specific displacer in fragment hit validation.

### Screening methodology

The principle behind the screening cascade is to prioritise those biophysical techniques that are quick, cheap and higher throughput and reserve the expensive and slower techniques for later in the triage process. This is guided by the desire to only start chemistry with well-validated hits. Initial screening results provide basic information about binding, with later screens having a much higher information content (frontline X-ray screening at Astex and HSQC NMR screening at Abbott being notable exceptions).[53–56] The consequence of this prioritisation is that, with each round of screening, the pool of compounds decreases, but confidence in the validity of the remaining hits increases. In the methodology presented here, an initial thermal-shift screen is used to identify a set of compounds that appear to bind to the protein and to disregard those that show no effect or destabilise the protein. Aggregating compounds can be spotted at this stage. Clearly, no information is gathered about binding location or target affinity and, taken in isolation, a small thermal shift is very weak validation of a hit. To build confidence in the hits, a much smaller, but enriched set of compounds is then taken forward into competitive STD NMR screening. Information about binding location can be gained through displacement experiments and an estimation of the *K*_d_ made from the degree to which a known binder is displaced. Although STD NMR screens are not immune to false positives from aggregating compounds, displacement experiments with a well-behaved displacer and control experiments with no protein should identify these compounds. The nature of the subsequent ITC experiments means they are much more resilient to false positives from compound aggregation. Therefore, this increasingly validated set can be taken through to ITC to quantify the binding constant directly at the expense of relatively large amounts of protein and time. Performing ITC experiments in competition mode also increases confidence in the binding pocket hypothesis. Finally, a collection of compounds that have shown good behaviour throughout the biophysical screening process is generated, and their structures in complex with the protein are then solved by X-ray crystallography. This provides the definitive evidence that the compound is well behaved and crucially identifies the mode of binding.

### Thermal-shift screening

The first step in the biophysical screening of the fragment library was a thermal-shift screen against the MAYSAM mutant of RadA. Briefly, the technique works by monitoring the thermal unfolding temperature (*T*_m_) of the protein by using an environmentally sensitive dye that fluoresces when the protein unfolds.[41] A compound that binds to and stabilises the protein will cause an increase in the melting temperature. The thermal shift, Δ*T*_m_, is calculated as the difference between the *T*_m_ of the protein incubated with the fragment and the *T*_m_ of the apo-protein control. A total of 1249 fragments were screened against the humanised MAYSAM RadA protein in 96-well plates. One fortuitous consequence of introducing the six humanising mutations was to lower *T*_m_ from >95 °C for the wild-type RadA to 81.0 °C for MAYSAM RadA, thus facilitating thermal-shift screening as complete denaturation curves can be recorded. A positive shift in the melting temperature of the protein in the presence of a fragment was interpreted as the formation of a more stable protein:ligand complex. One advantage of thermal-shift screening is that negative thermal shifts indicate fragments that denature the protein and so can be rejected from further analysis.[57] In kinetic- or competition-based assays, such as fluorescence polarisation with labelled reporter, such compounds would appear as false positives.

**Figure 3:**
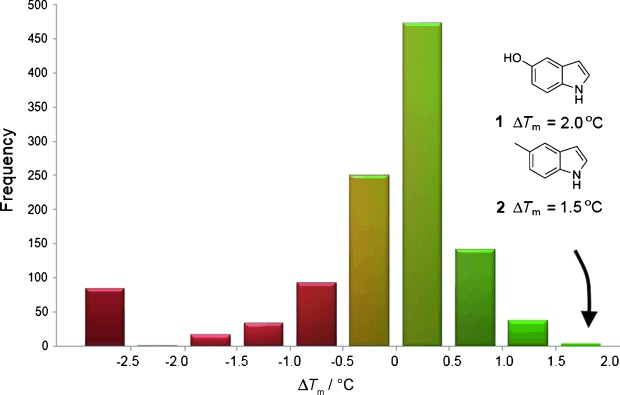
Histogram of the results of thermal-shift fragment screening with MAYSAM RadA. Fragments **1** and **2** induced the highest stabilisation.

From the fragments screened, 96 compounds were disregarded during the analysis as they produced complex thermal melting profiles, presumably arising either from optical interference in the assay or compound insolubility and aggregation problems. The majority of fragments, 60 %, caused no significant shift (between −0.5 and +0.5 °C) of the protein *T*_m_, and 22 % gave a Δ*T*_m_ more negative than −0.5 °C. A shift of greater than 1 °C (two standard deviations) was considered as a hit. Ranking these hits by Δ*T*_m_, the top two fragments were found to share a common indole core. These were 5-hydroxyindole (**1**) and 5-methylindole (**2**), which gave thermal shifts of +2.0 and +1.5 °C, respectively (Figure 3).

### Biophysical fragment validation of fragments 1 and 2

**Figure 4:**
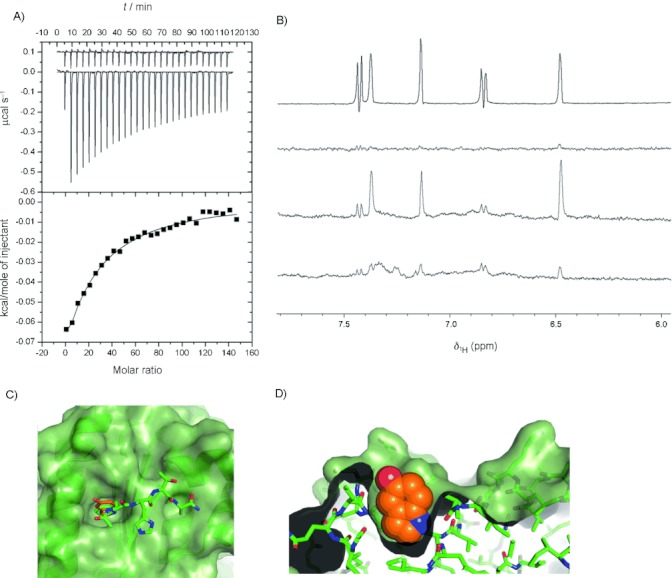
Biophysical validation of fragment hit 5-hydroxyindole **1** by A) ITC, *K*_d_=1.9 mm and B) STD NMR spectroscopy; from top: ^1^H NMR of aromatic region of 5-hydroxyindole, STD experiments in the absence of protein, with MAYSAM RadA and upon addition of 100 μm oligomerisation peptide. C) Crystal structure of **1** (orange) bound in the phenylalanine pocket of MAYM RadA, with the FHTA peptide (green) overlaid. D) Cross-section through the FXXA binding site with **1** shown as a space filling model.

The ability of the fragments **1** and **2** to bind to the MAYSAM RadA protein was demonstrated by saturation transfer difference (STD) NMR spectroscopy experiments. For example, fragment **1** produced no proton signals in an STD NMR spectrum in the absence of protein but produced strong signals in the presence of protein, thus indicating binding (Figure 4). These signals were greatly reduced upon addition of the RadA N-terminal oligomerisation peptide (Ac-NLGTFMRADEYLKKR-NH_2_, *K*_d_ against MAYSAM RadA=3.3 μm, Figure S1 in the Supporting Information), thereby suggesting competition with a common binding site. To quantify the binding of each fragment, the dissociation constant *K*_d_ was measured by ITC. Both **1** and **2** were found to bind with a *K*_d_ of 2.1 mm (Figures 4 and S2). These are typical affinities for fragment binding; their ligand efficiencies of 0.36 kcal mol^−1^ were also encouraging. In order to provide further evidence for the binding site of the fragments, competition experiments were performed by ITC. A titration of FHTA against protein preincubated with 5 mm fragment showed no binding of the peptide; this suggests competition between the two ligands. Conversely, a titration of ATP against protein preincubated with 5 mm fragment showed no effect on ATP binding at the distal ATP binding site (Figure S3). These results together with the thermal shifts and STD NMR spectroscopy experiments provide evidence that the fragments are behaving competitively with the FHTA ligand for the protein binding site, and not behaving as general denaturants. To gain further insight into the binding of the fragments, both were soaked into crystals of humanised MAYM RadA.

The 5-hydroxyindole fragment **1** is found buried in the hydrophobic phenylalanine pocket, packing particularly closely against the side chains of Met169, Ala218 and Met221 (Figure 4 C and D). Towards the base of the pocket, the indole NH forms a hydrogen bond with the backbone carbonyl of Ala201. The 5-hydroxy group makes two hydrogen bonds to the side chain of Gln217 and a water molecule coordinated to the backbone of Met221 and Gln217. The fragment is anchored at both ends by hydrogen bonds, with the indole scaffold contributing hydrophobic interactions with the pocket in addition to rigidly presenting both the indole NH and hydroxyl group to the protein in the suitable geometry. Interestingly, replacement of the 5-hydroxy group by a methyl group, as in fragment **2**, makes the indole core flip in the pocket to orientate the methyl group away from the water molecule, which remains bound in the crystal structure by residues Met221 and Gln217, rather than being displaced. The indole nitrogen proton forms a hydrogen bond with the backbone carbonyl group of Leu214.

### STD NMR screening

**Figure 5:**
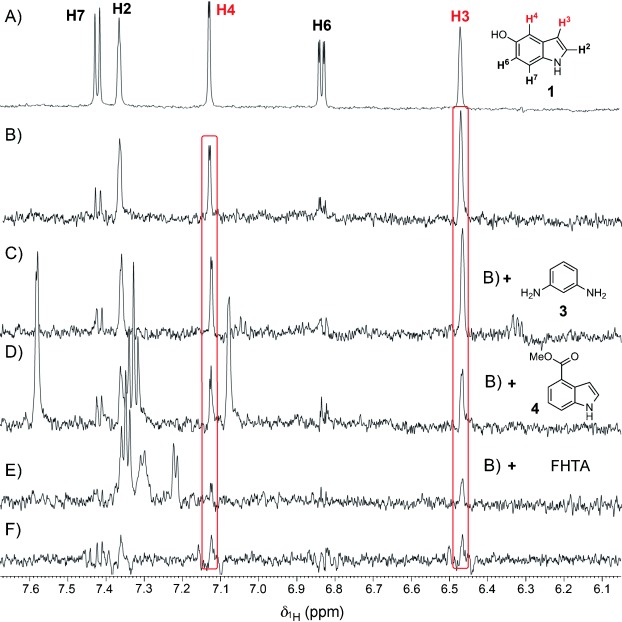
Example results from the first STD screen. A) ^1^H NMR spectrum of “reporter” ligand **1**. STD spectra of **1** in the presence of MAYSAM RadA protein (B) and in competition with C) 3-amino aniline **3**, D) 4-methyl ester indole **4** and E) tetrapeptide FHTA. F) Control STD spectrum of **1** in the absence of protein.

Ideally when advancing a fragment programme, it is desirable to identify a collection of fragments that present a variety of chemical scaffolds, vectors for fragment growth and physico-chemical properties. Having characterised the binding of the indoles identified by thermal shift as specifically accessing the surface phenylalanine pocket of humanised MAYSAM RadA, a competitive STD NMR screen was devised to identify further fragments that competed directly for this pocket. It was decided to use 5-hydroxyindole (**1**) as a “reporter” ligand and to monitor the STD NMR signals in the presence of the humanised protein, and the degree to which the candidate fragments could compete with the Phe pocket by the decrease in the STD signals of **1**. The tetrapeptide FHTA was also considered as a reporter molecule, but the coupled proton system of the phenyl moiety gave a weaker spectrum in STD experiments than did **1**. The H-3 and H-4 proton signals of the reporter molecule were largely distinct from the competing fragment peaks and could be monitored conveniently (Figure 5). A clear advantage of performing this STD screen as a series of competitive experiments with a known fragment **1**, rather than as direct STD experiments of candidate fragments to apo-protein, is that the degree of displacement of **1** allows the relative affinity of the competing fragment to be easily ranked and a crude estimate of *K*_d_ to be made.[58] It would not have been possible to confidently rank hits from a STD screen performed without a reporter ligand due to the variability of STD response arising from the compound-dependent kinetics of binding, binding mode and spin diffusion properties. In addition, the competitive STD screen answers a specific question about the location of binding, which was of interest, as well as defining the threshold of potency to find compounds that were at least comparable to, if not better than, the reporter ligand in terms of potency. The use of a well-behaved reporter ligand that can be cleanly displaced and recording STD spectra for every experiment in the absence of protein allows aggregating compounds to be readily identified. Finally the experiments potentially allow the detection of cooperatively binding fragments or fragments bound in other pockets by observing the emergence of new signals corresponding to the screened fragment but with no concomitant change in the STD signals of **1**. A small library of 42 compounds was selected for STD screening; it comprised fragments designed to investigate structure–activity relationships around the indole core and other fragments that showed a weakly positive Δ*T*_m_ of less than 1.0 °C in the prior thermal-shift screen. Each compound was screened at 1 mm as a singleton in the presence of fragment **1** and the humanised MAYSAM RadA protein. Control competitive STD experiments with FHTA demonstrated that the binding of indole **1** was completely abrogated by the tetrapeptide to baseline levels, similar to the control spectrum obtained in the absence of protein.

Most fragments screened had no significant effect on the signals of ligand **1**, for example, 3-aminoaniline (**3**, Figure 5 C). One of the most potent hits, 4-methylester indole (**4**), caused a 60 % decrease in the H-3 signal of **1**, and new peaks indicating binding of **4** were also observed (Figure 5 D). Indazole **5** was seen to produce a 55 % decrease in the H-3 signal of **1** (data not shown). In all cases the controls of the fragment singletons and **1** showed no significant STD response in the absence of protein. The two fragments identified from this STD screen (hit rate ≍5 %), **4** and **5**, were confirmed by ITC and the *K*_d_ values measured as 1.3 and 1.0 mm, respectively, Table 1.

**Table 1:** Summary of fragments identified from the screening programme by a methodology pipeline of thermal shift and STD NMR screening

	Compound	Screening technique	*K*_d_ [mm]	LE[a]	LLE[b]
	Ac-FHTA-NH_2_	–	0.21	0.14	6.9
**1**	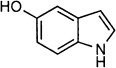	TS	2.1	0.36	1.5
**2**	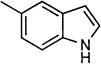	TS	2.1	0.36	0.6
**4**	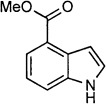	STD NMR[c]	1.3	0.30	1.5
**5**	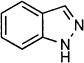	STD NMR[c]	1.0	0.46	1.2
**6**	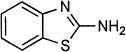	STD NMR[d]	0.73	0.43	0.7
**8**	l-methyl ester tryptophan	STD NMR[d]	0.57	0.28	2.7
**9**	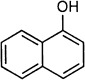	STD NMR[d]	0.43	0.42	0.5
**10**	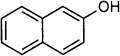	STD NMR[d]	0.46	0.41	0.7

[a] Ligand efficiency.

[b] Lipophilic ligand efficiency. c log *P* calculated in ChemBioDraw Ultra 12.0.

[c] Screen performed with fragment **1** as a reporter ligand.

[d] Secondary screen performed with fragment **4** as a reporter ligand.

**Figure 6:**
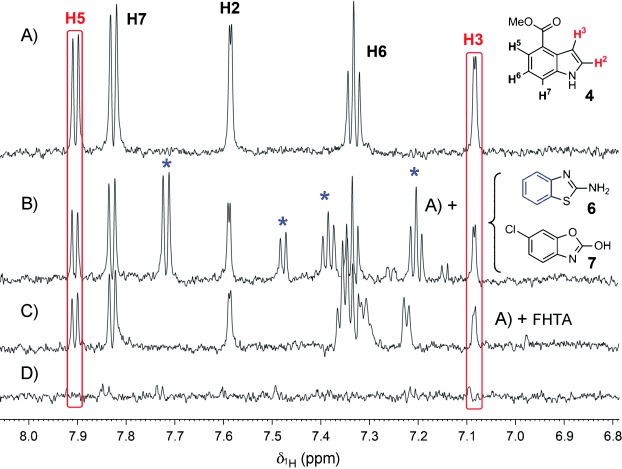
Example results from second STD screen. A) STD spectra of **4** in the presence of MAYSAM RadA protein. B) Displacement of **4** and binding of fragment **6** upon addition of a cocktail comprising 2-aminobenzothiazole (**6**) and 2-amino-6-chlorobenzoxazole (**7**). Blue asterisks indicate new signals from binding of fragment **6**. C) Control displacement with tetrapeptide FHTA. D) Control STD spectrum of **4** with cocktail and no protein.

Following on from the initial STD screen, a second competitive STD screen was performed with a larger set of fragment-like compounds. 4-Methyl ester indole (**4**) was chosen as the reporter ligand rather than fragment **1**. The increased potency of **4** as compared to **1** sets the dynamic range of the experiment such that the potency required to displace **4** is increased, excluding more weakly binding fragments. In total, 120 compounds, selected from a combination of in silico screening and inspection of commercially available fragment-like compounds, were screened in 60 cocktails of two. The H-3 and H-5 protons of **4** were distinct from observed new STD signals and could be monitored to quantify the percentage displacement of **4** in order to rank the cocktails. As an example, the cocktail comprising 2-aminobenzothiazole (**6**) and 2-amino-6-chlorobenzoxazole (**7**) produced a 40 % decrease in the STD signals of **4** and the appearance of peaks corresponding to 2-amino benzothiazole (**6**, Figure 6 B). The identification of **6** as the active component was confirmed by performing ITC titrations with each compound against the humanised protein. A *K*_d_ of 730 μm was measured for fragment **6**, but binding was not observed for **7**; this is consistent with the results of the NMR screen (Figure S4). In each case, deconvolution of cocktail hits to identify the active component was achieved by individual ITC titrations of the cocktail members against protein. In total, four new fragments were identified in this second STD screen: **6**, l-methylester tryptophan (**8**), naphth-1-ol (**9**) and naphth-2-ol (**10**).

## Discussion

**Figure 7:**
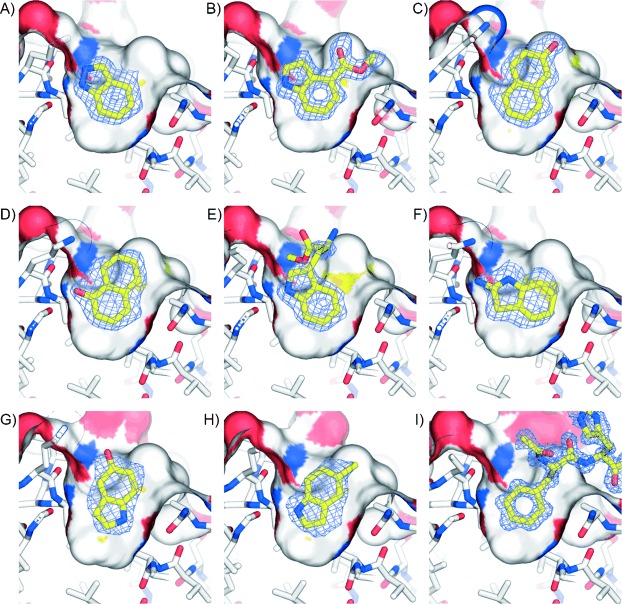
Side view through the Phe pocket of crystal structures of validated fragments and FHTA in complex with MAYM RadA A) Indazole (**5**), B) 4-methylester indole (**4**), C) naphth-1-ol (**9**), D) naphth-2-ol (**10**), E) l-methylester tryptophan (**8**), F) 2-aminobenzothiazole (**6**), G) 5-hydroxy indole (**1**), H) 5-methyl indole (**2**), I) FHTA tetrapeptide. Weighted 2*mF*_o_−*DF*_c_ electron density maps of the partially refined structures before inclusion of the ligands.

For each fragment identified and validated by this methodology, a crystal structure in complex with the humanised MAYM RadA protein was solved to confirm the binding of that fragment in the Phe pocket (Figure 7). With the exception of fragment **10**, all of the fragments identified by the STD NMR screen make a hydrogen bond with the backbone carbonyl of Leu214, in a similar fashion to indole **2**. From the crystal structure of **9** bound to the protein, it is clear that the hydroxy substituent at the 2-position of fragment **10** would not be tolerated in the bottom of the pocket if **10** bound in an analogous manner to **9**. Instead **10** is positioned with the hydroxy group projecting to the solvent, in a vector towards the alanine pocket. Indazole **5** forms a hydrogen bond with the side chain of Gln217, in addition to the interaction with Leu214. The methyl group of the ester of **8** displaces a water molecule bound to Met221 and Gln217 that is normally present in apo and other liganded structures and might contribute a favourable entropic term to its binding energy.

An overlay of the FHTA and fragment complexes with MAYM RadA reveals that the majority of side chains and backbone atoms in the vicinity of the phenylalanine pocket show little variation in conformation. However, the side chain of Gln217 adopts a variety of conformations. Comparing the crystal structures of FHTA and **6** bound to humanised RadA, the Cα of Gln217 is seen to shift by 1.7 Å, bowing the α-helix slightly and widening the Phe pocket to allow **6** to bind and make hydrogen bonds to both Leu214 and Gln217. It is not known what the energetic costs to the binding energy of such side chain and helix movements are; however it is supposed that a single conformation is induced by the ligand from a distribution of relatively low energy conformers of this side chain. Interestingly, the six-membered ring of the indole fragments and indazole superimpose very well with the most buried ring of the naphthol fragments. Only fragment **6**, in which the shape of the Phe pocket is changed, is not superimposable in this set of fragments; this potentially offers different vectors for chemical elaboration to the other fused 5–6 bicyclic heterocycles identified. In general, the high degree of structural overlap of the fragment scaffolds might mean that a successful strategy to grow one fragment could be expected to be analogously applied to another fragment.

In the crystal structures in complex with the fragments, a water molecule mimics the tetrapeptide by forming hydrogen bonds to the backbone amide of Tyr202 and is displaced when FHTA binds. The tetrapeptide also forms other hydrogen bonds between the backbone alanine nitrogen and the carbonyls of Leu197 and Ile200. Therefore, one strategy is to target these interactions by growing out from the fragment. A small hydrophobic group might then be positioned in the alanine pocket. There might also be potential for a π-stack on the phenolic ring of Tyr202 or interaction with the His210 side chain.

### Thermodynamic analysis of fragment binding

The *K*_d_ values determined by ITC, the thermodynamic parameters and the corresponding ligand efficiencies (LE) and lipophilic ligand efficiencies (LLE) for FHTA and all the fragments identified are summarised in Table 1. For these weak affinity ligands, it has been proposed that reliable measurements of Δ*G* can be obtained by ITC but that estimates of Δ*H* and therefore Δ*S* must be treated with some caution.[59] An enthalpy/entropy compensation effect is observed for the fragments, as increases in Δ*H* appear partly offset by increases in −*T*Δ*S*. It is therefore difficult to interpret and relate the structural information to the thermodynamic and *K*_d_ data. Interestingly, the FHTA tetrapeptide has a comparable Δ*H* to fragments **1** and **2** but a favourable entropic contribution to binding, possibly from displacement of surface-bound waters around the alanine pocket.

Potency, LE and LLE are useful drivers in deciding which fragments to chemically elaborate. Ligand efficiency is defined as the Gibbs free energy of binding divided by the number of non-hydrogen atoms.[60] A high LE is preferred, typically greater than 0.3. Polar fragments to which lipophilic groups can be attached are also favoured, so as to ensure the best chance that an elaborated fragment series does not become too hydrophobic. The LLE metric attempts to capture some penalty for hydrophobicity by subtracting log *P* from the p*K*_d_ or pIC_50_, a higher LLE value indicating more potency for less lipophilicity.[61] It is interesting to note that, of the fragments identified, tryptophan derivate **8** has the lowest LE (0.28) and possesses the highest LLE of 2.7. Conversely, solely on potency and LE, naphthols **9** and **10** look attractive but score poorly on LLE. On this basis alone, the synthetic elaboration of fragment **8** to a lead series might be more tractable than that of naphthols **9** and **10**.

## Conclusions

We have performed a thermal-shift fragment screen by using a surrogate protein designed to emulate the interaction site of the BRC4 repeat of human BRCA2 on RAD51. We have been able to identify efficient low-molecular-weight fragments that bind on the protein surface at the FHTA interaction site. The very low hit rate of ∼0.2 % in the thermal shift screen, as compared to similar screens conducted against enzyme active sites, is indicative of the challenges inherent to this type of target site. The top two hits from the screen were validated by biophysical techniques, and the crystal structures in complex with the protein were solved; these suggested suitable vectors for future chemical elaboration.

One of the fragments identified, 5-hydroxyindole (**1**), was subsequently used as a probe in a competitive STD NMR screen that identified other fragments competitive for the Phe pocket and enabled the relative potency of the fragments to be ranked. This provided a further small-molecule probe for a second, larger NMR screen. A panel of fragments was identified and further validated by ITC, and crystal structures in complex with the target protein were determined. In retrospect, it is noteworthy that the initial fragment **1** showed specific binding behaviour in STD NMR experiments and that such a modestly potent fragment could be used effectively to identify further hits.

All the fragments identified were found to bind in a small pocket on the surface of the protein in a fragment “hotspot” that naturally accommodates only the benzene ring of phenylalanine in the FXXA motif. For such a shallow binding pocket, it is somewhat surprising that the discovered fragments were able to bind with typical fragment affinities of low mm and that they possessed good ligand efficiencies of between 0.28–0.46, thus providing promising starting points for fragment elaboration. For these reasons, it is encouraging that other targets with similarly shallow binding sites might also be amenable to fragment screening. The use of a stabilised protein surrogate might also be a useful general tool for generating stable monomers, particularly in the field of protein–protein interactions in which it might not always be possible to study and screen unpartnered apo-proteins. The methodology of this screening and biophysical validation pipeline is hoped to be more generally applicable to interrogating other protein–protein interactions, and to expanding the toolbox of techniques available to identify fragments for this challenging and under-exploited set of targets. In conclusion, investment in a platform of orthogonal biophysical assays and screens is crucial for progression into a programme of medicinal chemistry. The elaboration of poorly validated hits not only has a high likelihood of failure, but without a variety of robust assays in place, the risk of being misled by badly behaving compounds increases.

### Significance

Protein–protein interactions remain a difficult and unexplored source of targets for future drugs. So far only a subset of these interactions has been considered druggable; they are based on deep hydrophobic α-helix-binding channels or clefts. In this work, however, we have taken inspiration from the interaction between the recombinase RAD51 and BRCA2, which is characterised by shallow phenylalanine and alanine binding pockets that interact with the conserved FXXA motif in interacting partners. A small molecule to abrogate this interaction could have applications in sensitising tumour cells to DNA damaging agents. By creating a stable monomeric form of RAD51 by humanising a related orthologue, a robust methodology of solution-based biophysical screens and X-ray crystallography was created to discover a series of heterocyclic fragment hits. Despite the shallow nature of the binding site, it was possible to identify several fragments that had potencies typical of fragments reported in the literature and that possessed very encouraging ligand efficiencies. Although this stage represents a starting point for fragment elaboration, future work should enable the development of these fragments into more potent lead molecules. In general, the ability to bind fragments in such shallow surface pockets adds confidence that similar PPIs can be successfully targeted by using fragment-based methods, thus expanding target space for small-molecule inhibition.

## Experimental Section

**Thermal denaturation assay:** The thermal-shift denaturation assay was performed on an iCycler iQ Real Time Detection System (Bio-Rad) in 96-well iCycler iQ PCR plates sealed with optically clear lids. The fluorescent dye Sypro Orange (a red-shifted dye used to minimise any optical interference from the fragments, Life Technologies Ltd., Paisley, UK) was used to report protein unfolding.[41] In a total volume of 100 μL, each well contained the MAYSAM RadA mutant (6.3 μm), 2.5× Sypro Orange, Tris (20 mm, pH 7.5) and NaCl (100 mm). Fragments were used at a final concentration of 5 mm in 5 % DMSO. Prior to use, the plates were spun for 2 min at 2000 rpm to remove air bubbles and solution from the lids. The plates were heated from 25 to 95 °C at a rate of 0.5 °C min^−1^. The fluorescence was monitored continuously with *λ*_ex_=490 nm and *λ*_em_=530 nm. To determine the thermal denaturation temperature, *T*_m_, for each well, the minimum of the negative derivative of the thermal melting curve was found. Δ*T*_m_ was calculated as the difference between the *T*_m_ of the protein/fragment mixture and that of a 5 % DMSO protein control.

**Isothermal titration calorimetry:** ITC experiments were performed at 25 °C on a MicroCal ITC-200 (GE Healthcare). Humanised RadA (600 μm) in 2-morpholinoethane sulfonic acid (MES, 20 mm, pH 6.0), NaCl (100 mm) and EDTA (0.5 mm) was diluted with Tris (200 mm, pH 7.5) containing NaCl (100 mm), and DMSO was added to match the ligand solution. Fragments in DMSO were diluted into the same buffer to give a final concentration of 10–15 mm ligand in 10 % DMSO and buffer. Care was taken to ensure that the DMSO concentrations in the protein and ligand solutions were well matched so as to avoid artefacts arising from heats of dilution of DMSO. In a typical experiment, protein (60 μm) was loaded in the sample cell, and 16 injections (2.4 μL) of 4.8 s duration were made at 80 s intervals from a syringe loaded with ligand (10–15 mm) and rotating at 1000 rpm. In all titrations, an initial injection of ligand (0.4 μL) was made and discarded during data analysis. Control titrations of ligand to buffer were performed and subtracted from the ligand-to-protein titrations. The thermodynamic parameters were obtained by fitting the data to a single-site-binding model by using Origin software and fixing the stoichiometry as 1.0 for weak binding ligands.[59, 62]

**Saturation-transfer difference NMR spectroscopy:** Samples for competitive STD NMR experiments were prepared typically with fragment(s) (1 mm) in 1 % DMSO, [D_4_]3-(trimethylsilyl)propionic acid as an internal standard (20 μm), D_2_O (10 %, *v*/*v*), with and without protein (20 μm). Each sample was made up to a final volume of 200 μL with Tris buffer (20 mm, pH 7.5) and NaCl (20 mm) in NMR tubes of 3 mm diameter (Hilgenberg, Malsfeld, Germany). A peptide concentration of 50 μm was used in displacement experiments with the RadA oligomerisation peptide. The experiments were performed on a Bruker Avance 700 MHz Ultrashield with a TXI cryoprobe equipped with a BACS-60 autosampler.

**Crystallography:** C-terminal ATPase domain of *P. furiosus* RadA (residues 108–349, with deletion of the “L2” loop residues 288–301) was expressed in the BL21(DE3) strain of *E. coli* under isopropyl-β-D-thiogalactopyranoside (IPTG)-inducible pBAT4 vector.[63] The soluble fraction of cells, lysed in MES (50 mm, pH 6.0), was heated at 65 °C for 10 min to denature most of the *E. coli* proteins. Soluble supernatant was purified on a 5 mL HiTrap Sepharose SP HP cation-exchange column at pH 6.0. The main peak fraction was concentrated and further purified by gel filtration on a Superdex 75 16/60 H*i*Prep column equilibrated with MES (20 mm, pH 6.0), NaCl (100 mm), and EDTA (0.5 mm). Pure protein was concentrated to 0.5 mm and flash frozen in liquid nitrogen. The MAYM RadA mutant was crystallised with 6–12 % PEG 1000 and Na/K phosphate (100 mm, pH 5.6–6.2) by the vapour-diffusion method. The fragments were soaked into the crystals of MAYM RadA for 15–20 h at 100 mm concentration in 8 % PEG 1000, Na/K phosphate (100 mm, pH 6.2), 20 % glycerol and 10 % DMSO. The crystals were flash frozen in liquid nitrogen in the same solution for later data collection. Data were collected at ESRF and Diamond synchrotron radiation sources, then processed by using the XDS package; structures were solved by using programs from the CCP4 package.[64] Models were iteratively refined and rebuilt by using Refmac5,[65] Phenix[66] and Coot[67] programs. Fragment coordinates were generated from SMILES strings by using the OpenEye software package, and restraints for crystallographic refinement were calculated by using the PRODRG program.[68] Coordinates have been deposited in the Protein Data Bank under accession codes 4b3c, 4d3d, 4b35, 4b2i, 4b34, 4b2L, 4b32, 4b33 and 4b3b. Data collection and refinement details are found in Table S1.

## Supplementary material

As a service to our authors and readers, this journal provides supporting information supplied by the authors. Such materials are peer reviewed and may be re-organized for online delivery, but are not copy-edited or typeset. Technical support issues arising from supporting information (other than missing files) should be addressed to the authors.
