# Supplementary material for: Using a Fragment-Based Approach To Target Protein–Protein Interactions
Source: Chembiochem. 2013 Jan 23;14(3):332–42. doi: 10.1002/cbic.201200521 (PMC3594973; doi:10.1002/cbic.201200521)
Supplement: Supplementary file 1 [file cbic0014-0332-SD1.pdf]

CHEMBIOCHEM

Supporting Information

© Copyright Wiley-VCH Verlag GmbH & Co. KGaA, 69451 Weinheim, 2013

Using a Fragment-Based Approach To Target Protein–Protein Interactions

Duncan E. Scott,[a] Matthias T. Ehebauer,[b, d] Tara Pukala,[a, e] May Marsh,[b] Tom L. Blundell,[b] Ashok R. Venkitaraman,[c] Chris Abell,[a] and Marko Hyvönen*[b]

cbic_201200521_sm_miscellaneous_information.pdf

Supplementary content

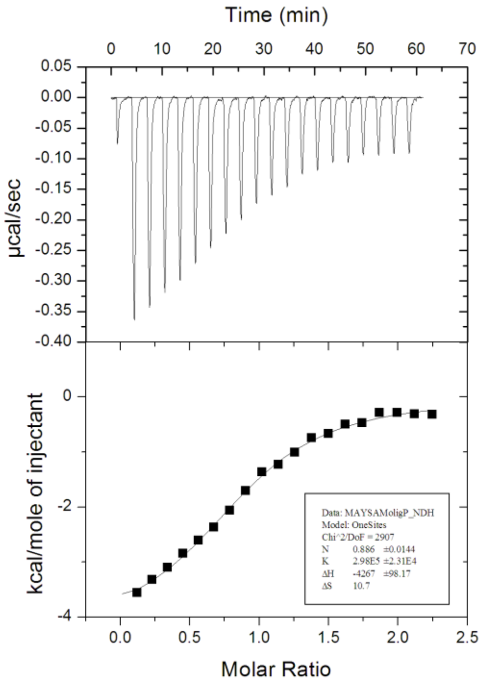

Figure S1 ITC of RadA oligomerization peptide Ac-NLGTFMRADEYLKKR-NH_2 against MAYSAM RadA, K_D = 3.3 μM

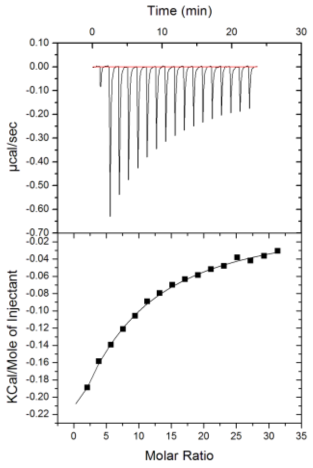

Figure S2 Titration of fragment **2** against MAYSAM RadA (K_D = 2.1 mM) .

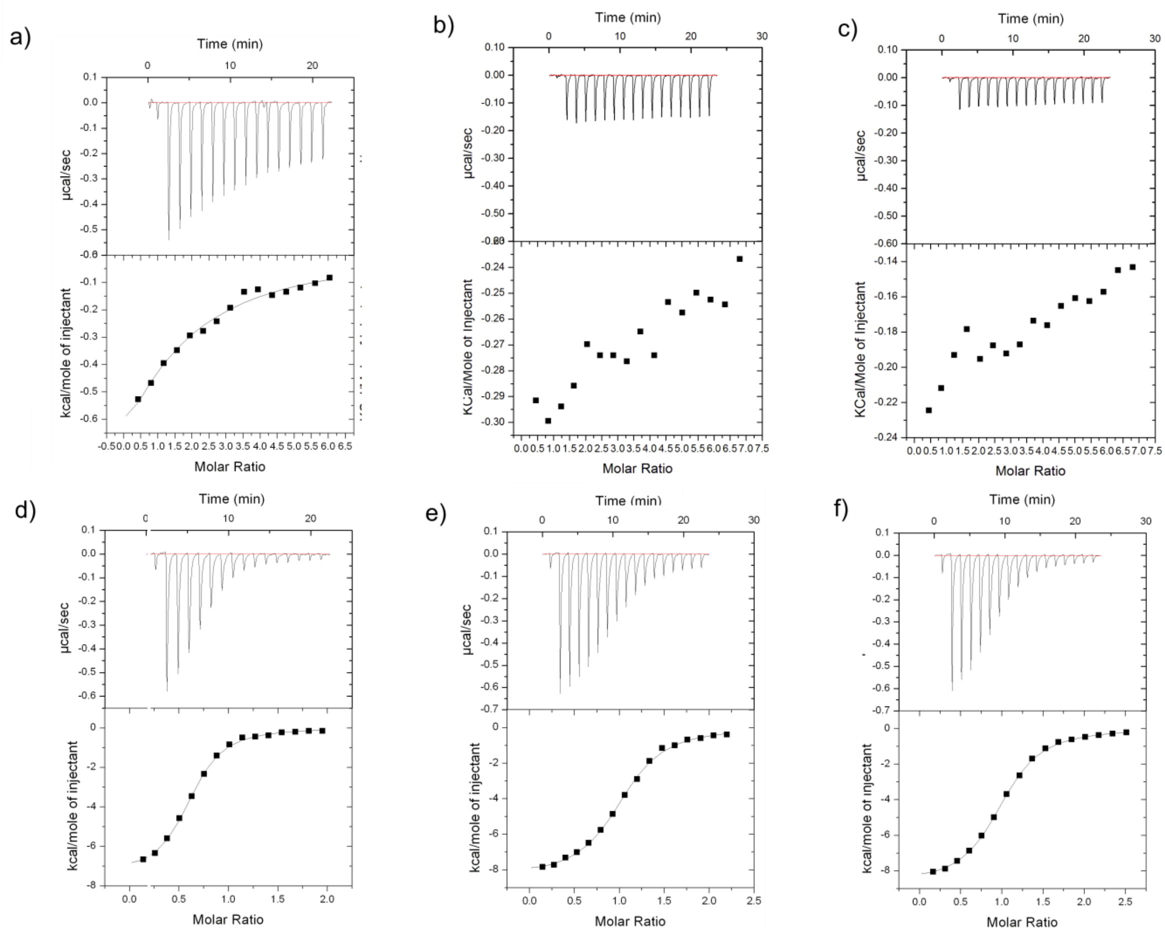

Figure S3 (a)-(c) Titrations of FHTA against (a) apo MAYSAM RadA (K_D = 250 $\pm$ 50 µM) (b) in the presence of fragment **1** (5 mM) and (c) in the presence of fragment **2** (5 mM). (d)-(f) Titrations of ATP against d) *apo* MAYSAM RadA (e) in the presence of fragment **1** (5 mM) and (f) in the presence of fragment **2** (5 mM).

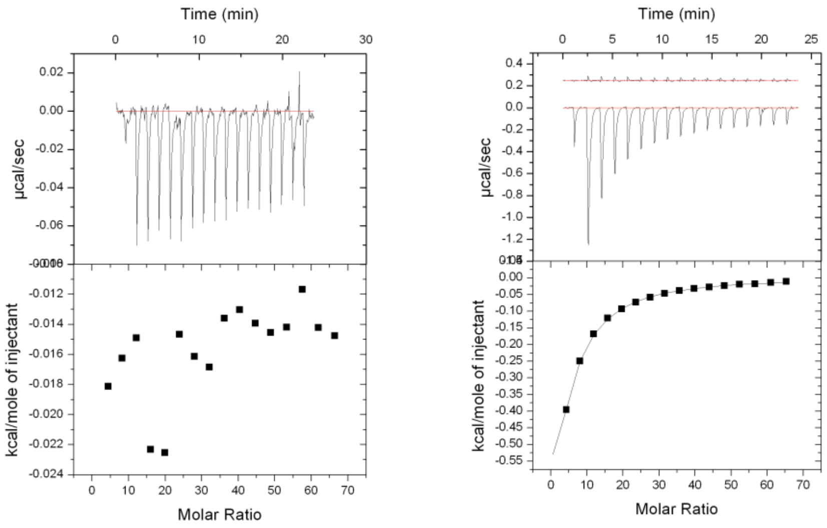

Figure S4 *Left:* Titration of 2-amino-6-chlorobenzoxazole **7** against MAYSAM RadA. *Right:* Titration of 2-aminobenzothiazole **6** against MAYSAM RadA, K_D = 0.73 mM

Supplementary Table S1, Scott et al.

Crystallographic data collection and refinement statistics (page 1)

Ligand	1 (5-hydroxy indole)	2 (5-methyl indole)	4 (4-methylester indole)	5 (indazole)	6 (4-amino benzothiazole)
PDB code:	4b3c	4d3d	4b35	4b2i	4b34
**Data collection:**					
Beamline:	In-house source	ESRF ID14-4	ESRF ID14-4	ESRF ID14-4	Diamond I04
X-ray wavelength:	1.5418 Å	0.9765 Å	0.9795 Å	0.9795 Å	0.9728 Å
**Data processing (XDS):**					
Resolution limit:	1.89 Å	1.59 Å	1.40 Å	1.30 Å	1.55 Å
High resolution shell:	2.00 - 1.89 Å	1.69 - 1.59 Å	1.48 - 1.40 Å	1.38 - 1.30 Å	1.64 - 1.55 Å
Completeness (high res. shell):	98.8% (93.0%)	99.2% (96.5%)	89.3% (91.7%)	98.8% (99.1%)	99.3% (98.8%)
No of unique reflections:	17821 (2653)	57763 (9076)	35882 (5917)	49540 (8001)	29702 (4763)
Rmerge:	11.7 % (73.5 %)	6.2 % (60.7 %)	3.6 % (45.8 %)	3.6 % (49.1 %)	5.2 % (58.0 %)
I/sigma(I):	14.65 (2.55)	13.76 (2.19)	16.16 (2.27)	18.21 (2.56)	16.49 (2.23)
Spacegroup:	P21212	P21	P21	P21	P21
Unit cell:	70.56 74.54 41.17 90.000 90.000 90.00	40.280 87.246 62.628 90.000 91.502 90	37.64 78.71 39.59 90.000 117.913 90.00	37.62 78.77 39.63 90.000 118.055 90.00	37.57 79.23 39.61 90.000 117.844 90.000
**Refinement (phenix.refine):**					
R factor	0.2072	0.1786	0.1896	0.1993	0.1868
R free	0.2612	0.2120	0.2231	0.2232	0.2236
msd bond lengts	0.010 Å	0.006 Å	0.006 Å	0.006 Å	0.006 Å
rmsd bond angles	1.186°	0.982°	1.508°	1.549°	1.566°
Ramachandran analysis % preferred / allowed regions	97.5/2.0	98.9/1.1	97/3	97/3	97/3

Crystallographic data collection and refinement statistics (page 2)

Ligand	8 (L-methyl ester tryptophan)	9 (napht-1-ol)	10 (napht-2-ol)	FHTA peptide
PDB code:	4b2l	4b32	4b33	4b3b
**Data collection:**				
Beamline:	Diamond I04	Diamond I04	Diamond I04	Diamond I04
X-ray wavelength:	0.972800 Å	0.972800 Å	0.972800 Å	0.9330 Å
**Data processing:**				
Resolution limit:	1.50 Å	1.50 Å	1.50 Å	1.19 Å
High resolution shell:	1.59 - 1.50 Å	1.59 - 1.50 Å	1.59 - 1.50 Å	1.26 - 1.19 Å
Completeness (high res. shell):	96.7% (94.6%)	99.2% (98.8%)	96.7% (95.2%)	93.4% (90.4%)
No of unique reflections:	31779 (4968)	32695 (5252)	31815 (5062)	64405 (9949)
Rmerge:	4.1 % (47.2 %)	4.2 % (37.6 %)	3.8 % (40.0 %)	5.8 % (24.0 %)
I/sigma (I):	19.16 (2.83)	18.36 (3.42)	19.18 (3.04)	16.06 (5.14)
Spacegroup:	P21	P21	P21	P212121
Unit cell:	37.60 78.63 39.62 90.000 117.479 90.000	37.69 79.05 39.62 90.000 117.883 90.000	37.66 78.71 39.67 90.000 117.906 90.000	40.225 60.589 87.526 90.000 90.000 90.000
**Refinement:**				
R factor	0.1862	0.1891	0.1882	0.1400
R free	0.2230	0.2276	0.2269	0.1671
msd bond lengts	0.006 Å	0.006 Å	0.006 Å	0.010 Å
rmsd bond angles	1.545°	1.577°	1.568°	1.397°
Ramachandran analysis % preferred / allowed regions	97.6/2.3	96.7/3.2	97.2/2.8	98.4/1.6
